# Controlling herding in minority game systems

**DOI:** 10.1038/srep20925

**Published:** 2016-02-17

**Authors:** Ji-Qiang Zhang, Zi-Gang Huang, Zhi-Xi Wu, Riqi Su, Ying-Cheng Lai

**Affiliations:** 1 Institute of Computational Physics and Complex Systems, Lanzhou University, Lanzhou Gansu 730000, China; 2 School of Electrical, Computer, and Energy Engineering, Arizona State University, Tempe, AZ 85287, USA; 3 Department of Physics, Arizona State University, Tempe, AZ 85287, USA

## Abstract

Resource allocation takes place in various types of real-world complex systems such as urban traffic, social services institutions, economical and ecosystems. Mathematically, the dynamical process of resource allocation can be modeled as *minority games*. Spontaneous evolution of the resource allocation dynamics, however, often leads to a harmful herding behavior accompanied by strong fluctuations in which a large majority of agents crowd temporarily for a few resources, leaving many others unused. Developing effective control methods to suppress and eliminate herding is an important but open problem. Here we develop a pinning control method, that the fluctuations of the system consist of intrinsic and systematic components allows us to design a control scheme with separated control variables. A striking finding is the universal existence of an optimal pinning fraction to minimize the variance of the system, regardless of the pinning patterns and the network topology. We carry out a generally applicable theory to explain the emergence of optimal pinning and to predict the dependence of the optimal pinning fraction on the network topology. Our work represents a general framework to deal with the broader problem of controlling collective dynamics in complex systems with potential applications in social, economical and political systems.

Resource allocation is an essential process in many real-world systems such as ecosystems of various sizes, transportation systems (e.g., Internet, urban traffic grids, rail and flight networks), public service providers (e.g., marts, hospitals, and schools), and social and economic organizations (e.g., banks and financial markets). The underlying system that supports resource allocation often contains a large number of interacting components or agents on a hierarchy of scales, and there are multiple resources available for each agent. As a result, complex behaviors are expected to emerge ubiquitously in the dynamical evolution of resource allocation. In particular, in a typical situation, agents or individuals possess similar capabilities in information processing and decision making, and they share the common goal of pursuing as high payoffs as possible. The interactions among the agents and their desire to maximize payoffs in competing for limited resources can lead to vast complexity in the system dynamics.

Given resource-allocation system that exhibits complex dynamics, a defining virtue of optimal performance is that the available resources are exploited evenly or uniformly by all agents in the system. In contrast, an undesired or even catastrophic behavior is the emergence of herding, in which a vast majority of agents concentrate on a few resources, leaving many other resources idle or unused^1^,^2^,^3^,^4^,^5^,^6^,^7^,^8^,^9^,^10^,^11^,^12^. Herd behavior has also attracted much attention in traditional economics^13^,^14^,^15^,^16^. If this behavior is not controlled, the few focused resources would be depleted, possibly directing agents to a different but still small set of resources. From a systems point of view, this can lead to a cascading type of failures as resources are being depleted one after another, eventually resulting in a catastrophic breakdown of the system on a global scale. In this paper, we analyze and test an effective method to control herding dynamics in complex resource-allocation systems.

A universal paradigm to model and understand the interactions and dynamical evolutions in many real world systems is complex adaptive systems^17^,^18^,^19^, among which minority game (MG)^20^,^21^ stands out as a particularly pertinent framework for resource allocation. MG dynamics was introduced by Challet and Zhang to address the classic El Farol bar-attendance problem conceived by Arthur^22^. In an MG system, each agent makes choice (e.g., + or −, to attend a bar or to stay at home) based on available global information from the previous round of interaction. The agents who pick the minority resource are rewarded, and those belonging to the majority group are punished due to limited resources. The MG dynamics has been studied extensively in the past^21^,^23^,^24^,^25^,^26^,^27^,^28^,^29^,^30^,^31^,^32^,^33^,^34^,^35^,^36^,^37^,^38^,^39^,^40^.

To analyze, understand, and exploit the MG dynamics, there are two theoretical approaches: mean field approximation and Boolean dynamics. The mean field approach was mainly developed by researchers from the statistical-physics community to cast the MG problem in the general framework of non-equilibrium phase transitions^21^,^20^,^41^,^42^. In the Boolean dynamics, for any agent, detailed information about the other agents that it interacts with is assumed to be available, and the agent responds accordingly^1^,^2^,^3^,^4^,^5^,^6^,^7^,^8^,^9^,^10^,^11^,^12^. Both approaches can lead to “better than random” performance in resource utilization. However, herding behavior in which many agents take identical action^43^ can also take place, which has been extensively studied and recognized as one important factor contributing to the origin of complexity that leads to enhanced fluctuations and, consequently, to significant degradation in efficiency^1^,^2^,^3^,^4^,^5^,^6^,^7^,^8^,^9^,^10^,^11^,^12^.

The control scheme we analyze in this paper is the pinning method that has been studied in controlling the collective dynamics, such as synchronization, in complex networks^11^,^44^,^45^,^46^,^47^,^48^,^49^,^50^. For the general setting of pinning control, the two key parameters are the “pinning fraction,” the fraction of agents chosen to hold a fixed state, and the “pinning pattern,” the configuration of plus or minus state assigned to the pinned agents. Our previous work^11^ treated the special case of two resources of identical capacities, where the pinning pattern was such that the probabilities of agents pinned to positive or negative state (to be defined later) are equal. Note that, while the pinned agents are frozen during system’s dynamical evolution, they are different from the “quenched” behavior in MG^23^. Especially, in our case the pinned states are a controlled state by design, but in typical MG dynamics the quenched behaviors are an emergent state through self organization. Here, we investigate a more realistic model setting and articulate a general mathematic control framework. A striking finding is that biased pinning control pattern can lead to an optimal pinning fraction for a variety of network topologies, so that the system efficiency can be improved remarkably. We develop a theoretical analysis based on the mean-field approximation to understand the non-monotonic behavior of the system efficiency about the optimal pinning fraction. We also study the dependence of the optimal fraction on the topological features of the system, such as the average degree and heterogeneity, and obtain a theoretical upper bound of the system efficiency. The theoretical predictions are validated with extensive numerical simulations. Our work represents a general framework to optimally control the collective dynamics in complex MG systems with potential applications in social, economical and political systems.

## Results

### Boolean dynamics

In the original Boolean system, a population of *N* agents compete for two alternative resources, denoted as *r* = + and *r* = −, which have the same accommodating capacity 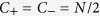. Similar to the MG dynamics, only the agents belonging to the *global minority* group are rewarded by one unit of payoff. As a result, the profit of the system is equal to the number of agents selecting the resource with attendance less than the accommodating capacity, which constitute the global-minority group. The dynamical variable of the Boolean system is denoted as 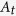, the number of + agents in the system at time step *t*. The variance of 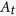 about the capacity 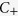 characterizes the efficiency of the system. The densities of the + and − agents in the whole system are 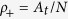 and 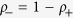, respectively. The state of the system can be conveniently specified by the column vector 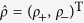.

A Boolean system has two states (a binary state system), in which agents make decision according to the local information from immediate neighbors. The neighborhood of an agent is determined by the connecting structure of the underlying network. Each agent receives inputs from its neighboring agents and updates its state according to the Boolean function, a function that generates either + and − from the inputs^3^. Realistically, for any agent, global information about the minority choice from all other agents at the preceding time step may not be available. Under this circumstance, the agent attempts to decide the global minority choice based on neighbors’ previous states. To be concrete, we assume^4^,^11^ that agent *i* with 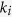 neighbors chooses + at time step *t* + 1 with the probability


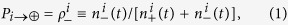


and chooses − with the probability 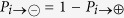, where 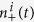 and 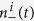, respectively, are the numbers of + and − neighbors of *i* at time step *t*, with 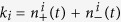. The expressions of probabilities, however, are valid only under the assumption that the two resources have the *same* accommodating capacity, i.e., 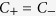. In real-world resource allocation systems, typically we have 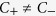. Consider, for example, the extreme case of 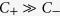. Suppose we have 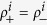 for agent *i*. In this case, rationality demands a stronger preference to the resource + (i.e., with a higher probability). To investigate the issues associated with the control of realistic Boolean dynamics, we define


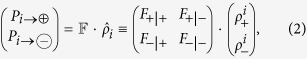


where 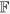 is the response function of each agent to its local environment 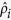, i.e., the local neighbor’s configuration with 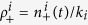 and 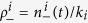. The quantity 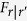 (or 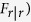 characterizes the contribution of the 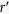-neighbors (or *r*-neighbors) to the probability for *i* to adopt *r*. The quantity 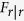 represents the strength of *assimilation* effect among the neighbors, while 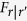 quantifies the *dissimilation* effect. Intuitively, the resource with a larger accommodating capacity would have a stronger assimilation effect among agents. By definition, the elements in each column in the matrix 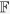 satisfy 
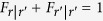
, i.e., the total probability for an agent to choose + and − is unity.

Using the mean-field assumption that the configuration of neighbors is uniform over the whole system, i.e., 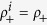, we have that the stable solution for Eq. (2) satisfies 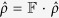, which leads to the eigenstate of 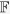 as


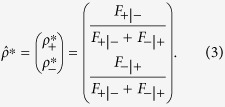


The rational response 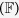 of agents to nonidentical accommodation capacities of resources will lead to the equality 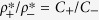, i.e., the stable fraction of the agent densities in + and − is simply the ratio of the capacities. The elements of 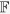 can then be defined accordingly using this ratio and the condition 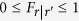, which characterizes a stronger preference to the resource with a larger capacity. For the specific case of identical-capacity resources, we have 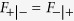, and the solution reduces to the result 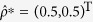 of the original Boolean dynamics^4^,^11^. The optimal solution for the resource allocation is 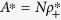.

A general measure of Boolean system’s performance is the variance of 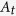 with respect to the *capacity* 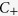:


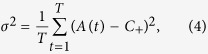


which characterizes, over a time interval 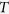, the statistical deviations from the optimal resource utilization^4^. A smaller value of *σ*^2^ indicates that the resource allocation is more optimal. A general phenomenon associated with Boolean dynamics is that, as agents strive to join the minority group, an undesired herding behavior can emerge, as characterized by large oscillations in 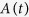. Our goal is to understand, for the general setting of nonidentical resource capacities, the effect of pinning control on suppressing/eliminating the herding behavior.

### Pinning control scheme

Following the general principle of pinning control of complex dynamical networks^11^,^44^,^45^,^46^,^47^,^48^,^49^,^50^, we set out to control the herding behavior by “pinning” a few agents to freeze their states during the dynamical evolution so as to realize optimal resource allocation for the entire network. Let 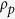 be the fraction of agents to be pinned, so the fraction of unpinned (or free) nodes is 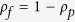. The numbers of the two different types of agents, respectively, are 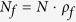 and 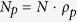. The free agents make choices according to local time-dependent information, for whom the inputs from the pinned agents are fixed.

The two basic quantities characterizing a pinning control scheme are the order of pinning (the way how certain agents are chosen to be pinned) and the pinning pattern^11^. We adopt the degree-preferential pinning (DPP) in which the agents are selected to be pinned according to their connectivity or degrees in the underlying network. In particular, agents of higher degrees are more likely to be pinned. This pinning method originated from the classic control method to mitigate the effects of intentional attacks in complex networks^51^,^52^,^53^. The selection of the pinning pattern can be characterized by the fractions 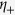 and 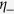 of the pinned agents that select 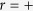 and 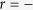, respectively, where 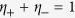. The quantities 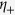 and 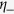 are thus the *pinning pattern indicators*. Different from the previous work^11^ that investigated the specific case of 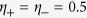 (half-half pinning pattern), here we consider the more general case where 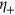 is treated as a variable. The pinning schemes are implemented on random networks and scale-free networks with different values of the scaling exponent *γ* in the power-law degree distribution^54^,^55^ 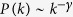. As we will see below, one uniform optimal pinning fraction 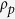 exists for various values of the pinning pattern indicator 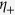.

### Simulation Results

**Figure 1:**
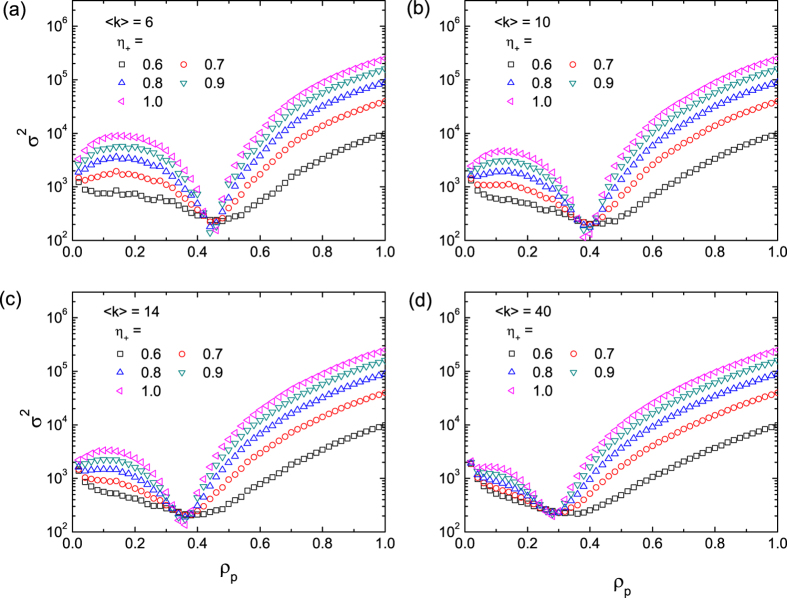
Variance *σ*^2^ as a function of the pinning fraction *ρ*_*p*_ for scale-free networks of different connection densities. The average degree of the networks for simulation are 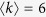, 10, 14, to 40 in (**a–d**), respectively, and the value of the pinning pattern indicator 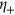 ranges from 0.6 to 1.0 for each panel. The results are averaged over 200 realizations for scale-free networks of size 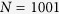 and degree exponent 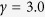. In each realization, the system evolves for 10000 time steps, and 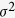 is calculated from the corresponding 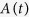, with the first 3000 time steps discarded to avoid the influence of transient state.

To gain insight, we first study the original Boolean dynamics with 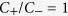 and 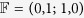 for different values of the pinning pattern indicator 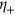. The game dynamics are implemented on scale-free networks of size 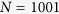 and of the scaling exponent 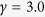
54 with the average degree 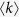 ranging from 6 to 40. The DPP scheme is performed with pinning fraction 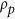 and 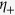 values ranging from 0.6 to 1.0 (i.e., all to + pinning). The variance 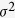 versus 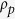 for different values of 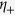 and different degree 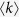 are shown in Fig. 1. We see that, in general, systems with larger values of 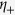 exhibit larger variance, implying that a larger deviation of 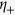 from the ratio of the capacity 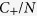 can lead to lower efficiency in resource allocation. Surprisingly, there exists a universal optimal pinning fraction (denoted by 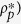 about 0.4, where the variance 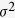 is minimized and exhibits an opposite trend for 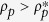, i.e., larger values of 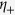 result in smaller values of *σ*^2^. The implication is that, deviations of 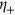 from 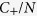 provide an opportunity to achieve better performance (with smaller variances *σ*^2^), due to the non-monotonic behavior of *σ*^2^ with 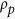. To understand the emergence of the optimal pinning fraction 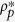, we see from Fig. 1 that the values of 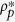 are approximately identical for different values of 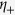, which decrease with the average degree 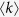. As we will see below, in the large degree limit 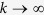, the value of *σ*^2^ can be predicted theoretically (c.f. Fig. 4).

**Figure 2:**
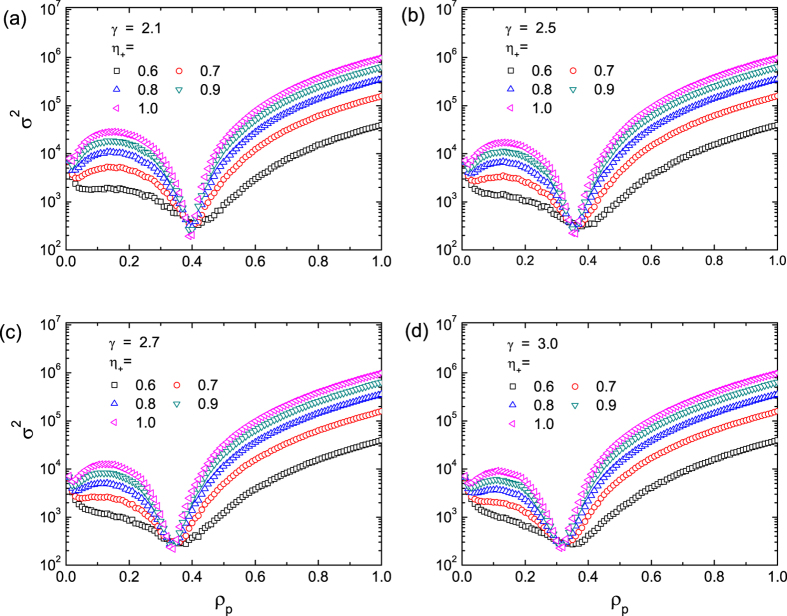
Variance *σ*^2^ as a function of the pinning fraction *ρ*_*p*_ for scale-free networks of varying degrees of heterogeneity. The scaling exponents of the networks are 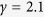, 2.5, 2.7, and 3.0 in (**a**–**d**), respectively, and the value of the pinning pattern indicator 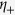 ranges from 0.6 to 1.0 for each panel. The results are averaged over 200 realizations for scale-free networks of size 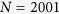 and average degree 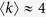. In each realization, the system evolves for 10000 time steps, and 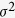 is calculated from the corresponding 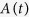, with the first 3000 time steps discarded to avoid the influence of transient state.

Simulations using scale-free networks of different degrees of heterogeneity also indicate the existence of the universal optimal pinning control scheme, as can be seen from the behaviors of the variance calculated from scale-free networks of different degree exponents (Fig. 2)^55^, where smaller values of *γ* point to a stronger degree of heterogeneity of the system. We see that an optimal value of 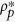 exists for all cases, which decreases only slightly with *γ*, i.e., more heterogeneous networks exhibit larger values of the optimal pinning fraction 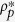, a phenomenon that can also be predicated theoretically (c.f. Fig. 5).

### Theoretical Analysis

The phenomenon of the existence of a universal optimal pinning fraction 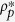, independent of the specific values of pinning pattern indicator 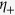, is remarkable. Here we develop a quantitative theory to explain this phenomenon.

**Figure 3:**
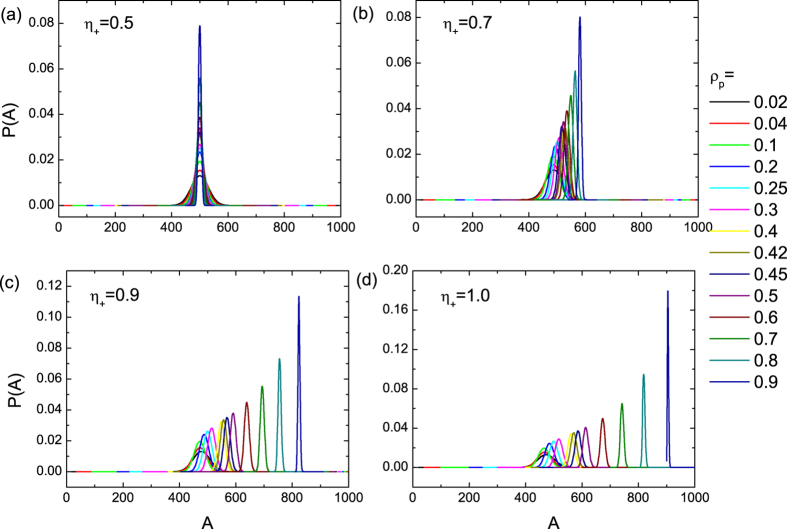
Theoretical prediction of the probability density distribution of attendance *A*. The distribution 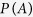 is obtained from the transition matrix Eq. (7) for 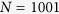. The value of the pinning pattern indicator 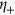 is set as 0.5, 0.7, 0.9 and 1.0 in (**a**–**d**), respectively, and the pinning fraction 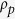 ranges from 0.02 to 0.9.

To begin, we note that MG is effectively a stochastic dynamical process due to the randomness in the selection of states by the agents. The variance of the system, a measure of the efficiency of the system, is determined by two separated factors. The first, denoted as 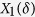, is the intrinsic fluctuations of *A* about its expected value 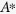, defined as 
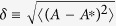
, which can be calculated once the stable distribution of attendance 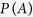 is known, where 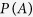 can be obtained either analytically (c.f., Fig. 3) or numerically. The second factor, denoted as 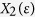, is the difference of the expected value 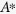 from the capacity 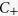 of the system: 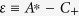, which also contributes to the variance of the system. Taking into account the two factors, we can write the system variance *σ*^2^ [defined in Eq. (4)] as


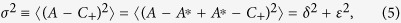


which is a sum of two factors: 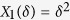 and 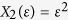. In contrast to the special case of 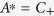 treated in previous works^4^,^11^, the more general cases are that the expected value 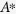 is not equal to the capacity 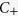. Nonzero values of 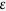 are a result of the biased pinning pattern (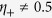) or improper response to the limited capacities of the resources. In fact, recent studies of the flux-fluctuation law in complex dynamical systems indicated that the variance of the system is determined by the two factors: intrinsic fluctuations and external driving^56^,^57^,^58^,^59^,^60^,^61^,^62^.

### Stable distribution of attendance

To quantify the process of biased pinning control, we derive a discrete-time master equation and then discuss the effect of network topology on control.

### Discrete-time master equation for biased pinning control

To understand the response of the Boolean dynamics to pinning control with varied values of the pinning pattern indicator 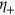, we generalize our previously developed analysis^11^. Let 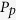 be the probability for a *neighbor* of one given *free* agent to be pinned so that the probability of encountering a free agent is 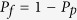. The transition probability of the system from 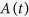 to 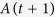 can be expressed in terms of 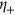. In particular, note that the state transition is due to updating of the 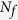 free agents, as the remaining 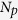 agents are fixed. To simplify notations, we set 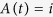, 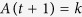, and 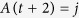, for 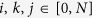. The conditional transition probability from *i* at *t* to *k* at *t* + 1 is


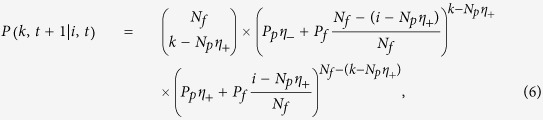


where 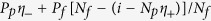 is the probability for a free agent to choose + with the first and second terms representing the contributions of the pinned − and free − neighbors, respectively. In the Boolean system, the values of attendance *A* oscillate about its equilibrium value^11^. The transition probability between the state at *t* and 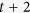 can be expressed as a function of 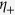:


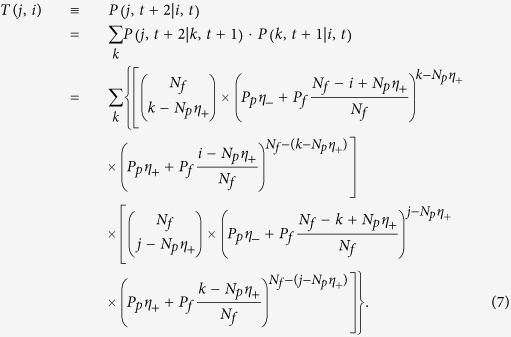


Equation (7) takes into account the effect of pinning patterns, which was ignored previously^11^. The resulting balance equation governing the dynamics of the Markov chains becomes


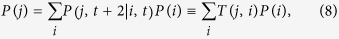


which is the discrete-time master equation. The stable state that the system evolves into can be defined in the matrix form as


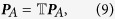


where 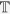 is an 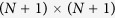 matrix with elements 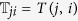, and 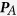 is the corresponding vector of 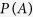 with *A* ranging from 0 to *N*.

The probability distribution 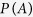 is a binomial function with various expectation values, as shown in Fig. 3. In addition, the probability 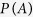 is zero for 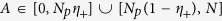, which defines the boundary condition in the sense that there are 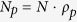 pinned agents. Once the stable distribution 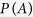 is obtained from Eq. (9), the cumulative variance of the system can be calculated from


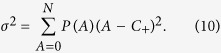


The theoretical prediction of 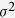 as a function of 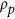 can thus be made through (*a*) identifying the function 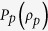, (*b*) defining the matrix 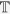 that depends on 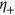 and 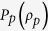, and (*c*) calculating the stable state 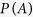.

### Effect of network topology on pinning control

The topology of the network system has an effect on the probability 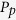. For the particular case of scale-free networks with degree exponent 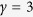, our previous work^11^ demonstrated that preferential pinning of the large-degree agents leads to 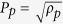. Here, we consider systems with degree distribution 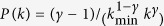, where 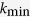 is the minimum degree of the network. For the DPP scheme where pinning occurs in the order from large to small degree agents, the relation between the minimum degree of *pinned* agents (denoted by 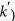 and the pinning fraction 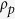 is


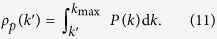


For a given pinning fraction 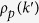 in which all the agents with 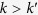 are pinned, the probability 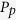 for one neighbor of a given *free* agent to be a pinned agent is given by


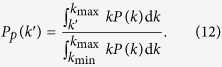


Here, Eqs. (11) and (12) are applicable to the DPP scheme on networks of any degree distribution 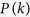 without degree correlation. The underlying assumption in Eq. (12) is that the degrees of the neighboring agents are not correlated, i.e., the neighbors of the pinned agents obey the same degree distribution 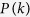 of the whole system. For a scale-free network, 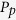 as a function of 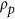 can be expressed as


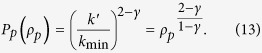


**Figure 4:**
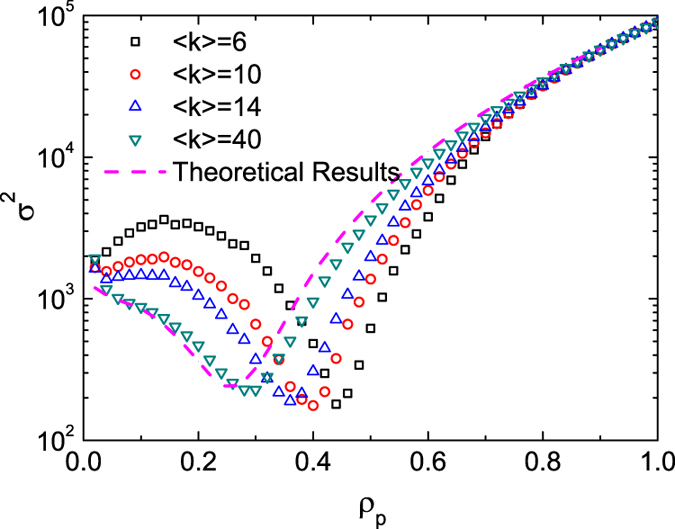
Theoretical prediction of the variance *σ*^2^ in comparison with the simulation results. The system has size 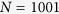 and power-law degree distribution 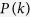 with scaling exponent 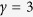. The theoretical prediction does not depend on the value of the average degree. In direct simulations, the values of the average degree are 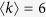, 10, 14, and 40. The simulation results denoted by symbols are the same as those plotted in Fig. 1, with the pinning pattern indicator to be 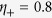.

For the special case of 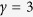, Eq. (13) reduces to the specific relationship obtained earlier^11^: 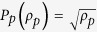. As indicated by Eqs. (7, the specific form of matrix 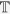 with respect to 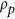 can be obtained by substituting Eq. (13) into Eq. (7), leading to the distribution 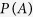 and finally the variance of the system 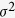 as a function of 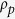. Figure 4 displays the theoretical predicted 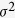 (dashed curves) for various values of the pinning fraction 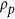 and of the pinning pattern indicator 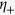. The trend and, more importantly, the existence of the optimal pinning fraction 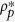, agree well with the simulation results (marked with different symbols). In the limit 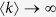, the system approaches a well-mixed state that can be fully characterized by Eq. (13), indicating that the simulation results approach the curve predicted by the mean-field theory as the average degree 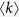 is increased.

**Figure 5:**
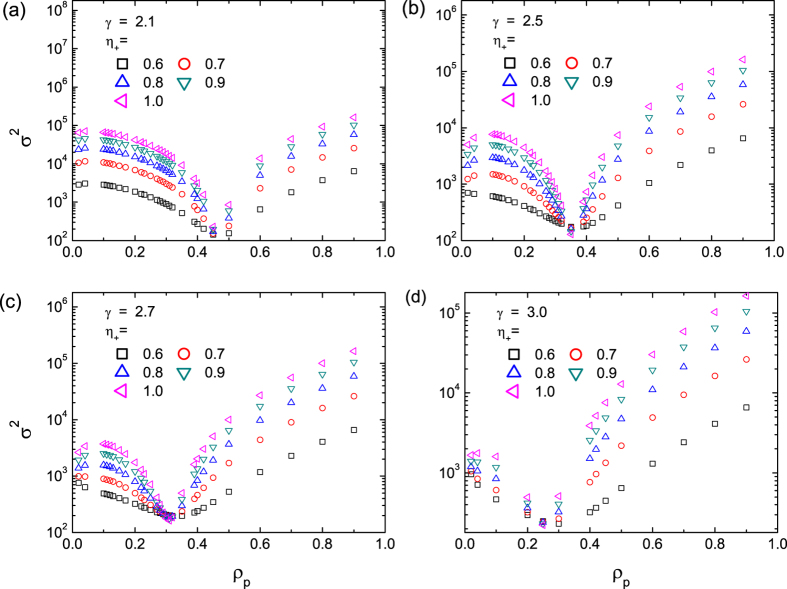
Theoretical prediction of variance *σ*^2^ for systems with different degree scaling exponents. The system has size 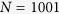 and power-law degree distribution 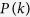 with different values of the degree exponent: (**a**–**d**) 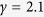, 2.5, 2.7, 3.0, respectively. In each case, the value of the pinning pattern indicator 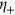 ranges from 0.6 to 1.0.

Figure 5 shows the theoretical prediction of 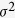 for scale-free networks with different values of the degree exponent *γ*, which agrees well with the results from direct simulation as in Fig. 2. For the case of highly heterogeneous networks 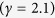, the theoretical prediction deviates slightly from the numerical results for the reason that the networks in simulation inevitably exhibit certain topological features that are not taken into account in the theoretical analysis of 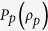, such as the degree correlation.

### Optimal pinning

Our analysis based on the master equation (8) applies to systems with 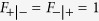 and identical resource capacity. We now consider the more general case of varying 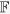 values to further understand the optimal pinning control scheme.

### Deviation of expected attendance from resource capacity

The dependence of 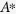 on 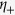 can be obtained through the general form of the response matrix 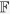. For convenience, we use the column vector 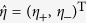 to denote the fraction of the agents pinned at + and −, where 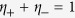, 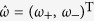 is the fraction of free agents adopting states + and −, respectively, with 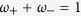. The state of the system can be expressed as 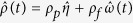, from which we have


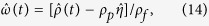


At the next time step, the expected value of the state based on 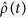 through the response matrix 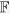 can be written as


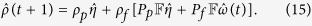


Substituting Eq. (14) into Eq. (15), we get the relationship between 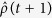 and 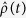. A self-consistency process stipulated by Eqs. (14) and (15) can yield the stable state of the system with the expected number of agents choosing + given by


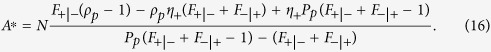


In a free system without pinning, the rational response 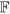 of agents to nonidentical capacities of resources leads to Eq. (3), implying the relationship 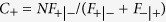. From Eq. (16), we can obtain *ε* as a function of the value of the pinning pattern indicator 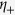, the elements of the matrix 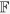, the pinning fraction 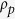, and the parameter 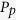 associated with network topology. We have


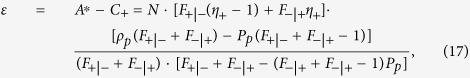


which has the form of separated variables associated with 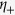 and 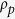.

### Optimal pinning pattern and fraction

Optimizing the system requires minimum 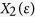, i.e., 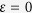 in Eq. (17), leading to two independent solutions:


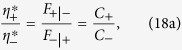



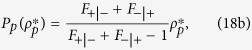


which respectively correspond to the optimal value of the pinning pattern indicator 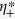 and the optimal pinning fraction 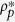. Here, for convenience, we define a parameter: 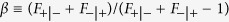 so that Eq. (18b) can be expressed concisely as 
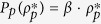
. Once the values of 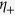 and 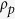 satisfy either Eq. (18a) or Eq. (18b), we can obtain 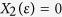. The variance 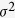 depends on the fluctuation factor 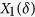 only.

Equation (18a) specifies the pinning pattern with the same ratio as that of the resource capacity. The Boolean dynamics studied previously^11^ is a special case where the optimal pinning pattern indicator is 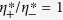 (i.e., 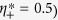 for systems with 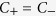, and the variance 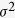 is simply determined by the factor 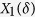 alone.

From Eq. (18b), we see that the optimal pinning fraction 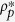 is independent of 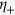 but depends on both the network structure through 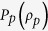 and on the response function 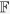. Additionally, the condition 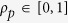 and nonzero denominator require


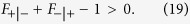


**Figure 6:**
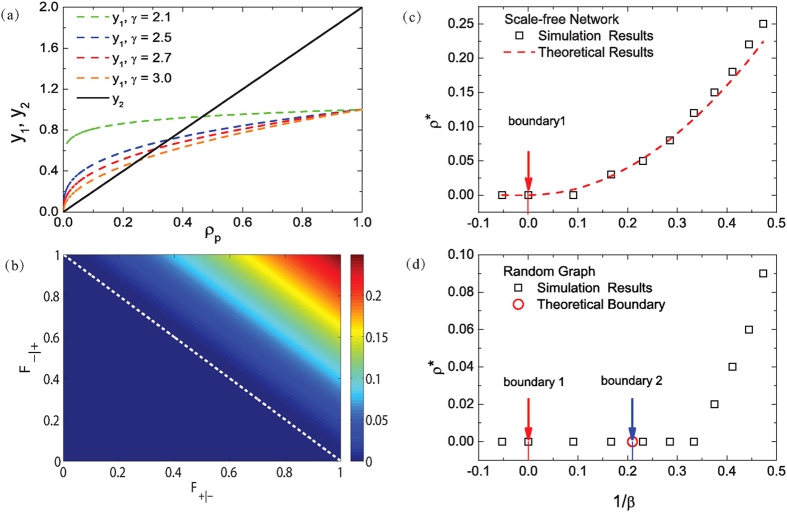
Optimal pinning fraction. (**a**) Intersections of the curves 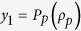 and 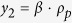 denote nonzero optimal pinning fraction 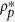 given by Eq. (18b). The scale-free networks have the degree exponents 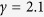, 2.5, 2.7, and 3.0, respectively. The response function is for 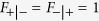 (corresponding to 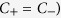. (**b**) Contour map of 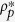 in the parameter space of 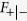 and 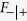 for scale-free networks with 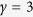. In the lower-left region below the boundary 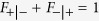 (white dashed line), nonzero solution of 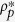 cannot be obtained. (**c**) Optimal pinning fraction 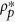 as a function of 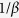 for scale-free networks. The analytical results from Eq. (18b) (red solid curve) and the simulation results (black open squares) agree well with each other. The red arrow marks the theoretical prediction of the boundary, where nonzero 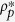 solutions exist on the left side. (**d**) For ER random networks, 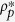 as a function of 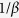. Theoretical results from Eq. (18b) (red open circle) and simulation results (black open squares) are shown. The boundaries 1 and 2 obtained theoretically (pointed to by solid arrows), respectively, stand for the constraint in Eqs. (19) and (22). In (**c**,**d**), the value of 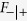 varies but 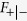 is set to 0.9. The scale-free and random networks used in the simulations have 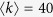 and 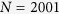.

The function 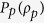 for scale-free networks, as in Eq. (13), increases monotonically with 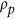. Figure 6(a) displays the curves 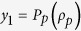 and 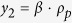, i.e., both sides of Eq. (18b). The existence of nonzero 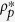 for 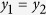 demands


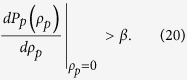


For scale-free networks, 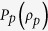 diverges at 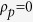. Equation (20) thus holds, implying that the DPP pinning scheme has a nonzero optimal pinning fraction 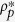, leading to 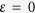. However, for homogeneous networks, Eq. (20) may not hold. In this case, a more specific implicit condition can be obtained from Eq. (20) through the following analysis. In particular, without an analytical expression of 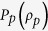, the derivative of 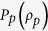 with respect to 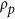 can be obtained from Eqs. (11) and (12):


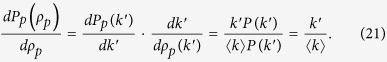


For degree preferential pinning, in the limit 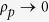, the maximum degree for *free* agents is 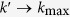. We thus have


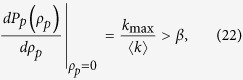


which requires that the network be heterogeneous. For 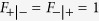, we have 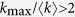, ensuring the existence of a nonzero 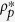 value for 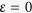.

The contour map of the optimal pinning fraction 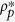 in the parameter space of 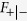 and 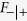 for scale-free networks with 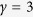 is shown in Fig. 6(b). The boundary 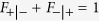 associated with condition Eq. (19) is represented by the white dashed line, where nonzero solutions of 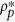 do not exist below the lower-left region. Figure 6(c,d) show 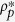 for 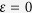 as a function of 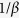 for scale-free and random networks, respectively, where 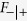 is varied and 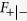 is fixed to 0.9. The theoretical prediction of 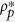 [red solid curve in (c) and red open circle in (d)] is given by the intersections of the curves 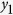 and 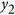 in Fig. 6(a). For scale-free networks, since Eq. (20) holds, Eq. (21) is the only constraint on the value of 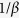 (red dashed arrow), with the region at the right-hand side yielding nonzero 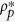 solutions. The red solid curve in Fig. 6(c) represents the theoretical prediction, and the open squares denote the simulation results from scale-free networks of size 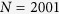, power-law exponent 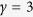, and average degree 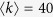.

For random networks, the existence of nonzero 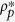 solutions requires that Eqs. (19) and (21) or (22) hold. For the Poisson degree distribution, the maximum degree of the network can be calculated from


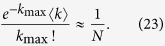


We can obtain an estimate of the value of 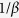 that satisfies Eq. (22), as indicated by the blue arrow (labeled as boundary 2) in Fig. 6(d). The right-hand side of this point satisfies both Eqs. (19) and (22), implying the existence of nonzero 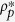. Comparison of the results from random and scale-free networks with different scaling exponents (Figs 2,5 and 6) shows that, stronger heterogeneity tends to enhance the values of 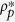, which can also be seen from Eq. (20).

To better understand the non-monotonic behavior of 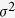 with 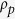, we provide a physical picture of the behavioral change for 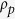 greater or less than 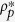. The effect of pinning control is determined by the number of edges between pinned and free agents, which are *pinning-free edges*. For a small pinning fraction 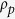, the average effect per pinned agent on the system (represented by the number of pinned-free edges per pinned agent) is relatively large. However, as 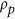 is increased, the average impact is reduced for two reasons: (*a*) an increase in the edges within the pinned agents’ community itself (i.e., two connected pinned agents), which has no effect on control, and (*b*) a decrease in the number of free agents, which directly reduces the number of pinned-free edges. Consider the special case of 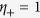 and 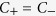. For small 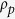, the pinned + agents have a significant impact so that the free agents tend to overestimate the probability of winning by adopting −. In this case, the expected value 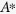 is smaller than 0.5*N*, corresponding to 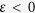. For highly heterogeneous systems, the average impact per pinned agent is larger for a given small value of 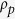. As 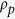 is increased, the average influence per pinned agent reduces and, consequently, 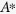 restores towards 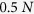. For 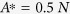 and 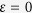, the system variance [Eq. (5)] is minimized due to 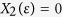, and the corresponding pinning fraction achieves the optimal value 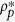. For strongly heterogeneous systems, due to the large initial average impact caused by pinning the hub agents, the optimal pinning fraction 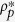 appears in the larger 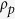 region. Further increase in 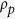 with 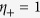 will lead to 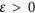 and 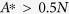, thereby introducing nonzero 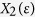 again and, consequently, generating an increasing trend in 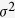.

### Collapse of variance

For certain networks, the variance 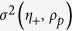 is determined by the values of the pinning pattern indicator 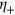 and the pinning fraction 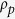. Our analysis so far focuses on the contribution of 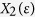 to the variance 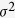 as the pinning fraction 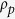 is increased but for fixed 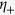. It is thus useful to define a quantity related to the variance 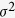, which can be expressed in the form of separated variables. For two different values of the pinning pattern indicator, 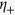 and 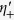, for a given value of 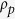, the relative weight of 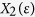 can be obtained from Eq. (17) as


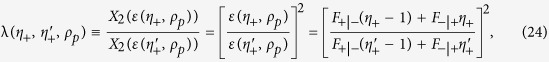


where 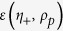 is a function of both 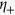 and 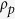. Remarkably, the ratio λ depends on 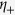 and 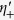 but it is independent of 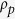, due to the form of separated variables in Eq. (17). From the simple relationship Eq. (24), we can define the relative changes in these quantities due to an increase in the value of 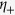 from a *reference value* 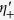 as


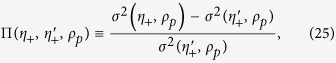



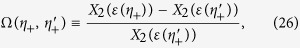


and then obtain the change rate associated with 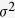 and 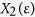 as,


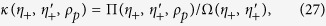


where 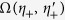 is independent of 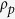. In the limit 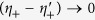, the rate of change 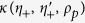 becomes


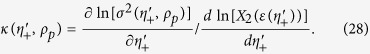


**Figure 7:**
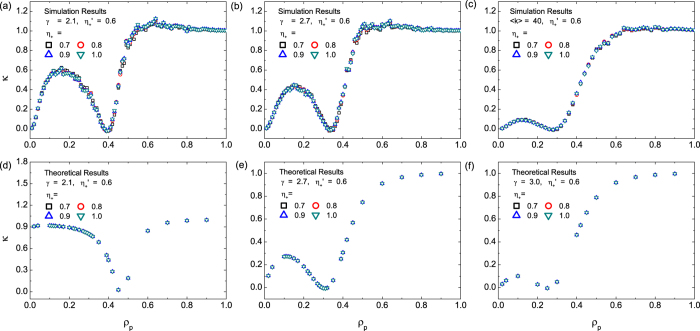
Collapse of *κ* for different pinning patterns. (**a**–**c**) Simulation results of 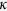 from scale-free networks for 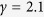, 2.7, and 3.0, which correspond to the results of 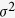 in Figs 1(d) and 2(a,c), respectively. (**d**–**f**) Theoretical results of 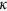 from Eq. (27) for the cases shown in Fig. 5(a,c,d), respectively. The reference pinning pattern indicator is 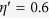.

Figure 7 shows 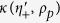 as a function of 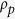 for scale-free networks, where the value of the reference pinning pattern indicator is 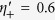. To obtain the values of 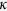, we first calculate Ω by substituting the values of 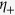, 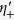 and the elements of 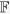 into Eqs. (24) and (26). We then obtain 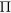 by substituting the values of 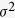 into Eq. (25), with 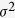 either from simulation as in Figs 1 and 2 or from theoretical analysis as in Fig. 5. We see that the 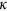 values from simulation results of 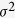 [Fig. 7(a–c) marked by “Simulation Results”] and theoretical prediction of 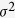 [Fig. 7(d–f) marked by “Theoretical Results”] show the behavior in which the curves of 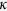 for different values of 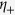 collapse into a single one. This indicates that 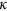 depends solely on the pinning fraction 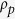; it is independent of the value of the pinning pattern indicator 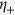. Extensive simulations and analysis of scale-free networks with different average degree 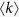 or different degree exponent *γ* verify the generality of the collapsing behavior.

From Eq. (28), we see that the variance 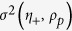 and the quantity 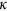 are closely related. For example, a smaller value of 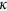 indicates that 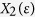 contributes more to the variance of 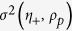 as 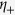 is changed, and vice versa. In Fig. 7, 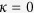 corresponds to the intersecting points of the curves of 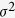 with different values of 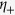 shown in Figs 1,2 and 5. It can also be verified analytically that, the minimal point with 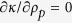 coincides with the optimal pinning fraction 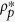 at which 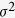 is minimized, which is supported by simulation results in Figs 1,2,5 and 7.

### Variance in the form of separated variables

**Figure 8:**
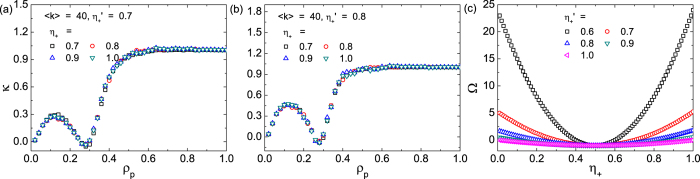
Two separated functions *κ* and Ω in Eq.(29). (**a**,**b**) Collapse of 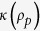 for various 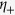 values, where the reference value is 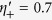 in (**a**) and 0.8 in (**b**). The values of 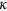 are predicted from Eq. (27) for a scale-free network with 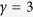 and 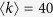. (**c**) The function 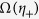 for 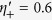, 0.7, 0.8, 0.9, 1.0, and *F*_+|−_ = *F*_−|+_ = 1.

From Eq. (27), for a given value of the reference pinning pattern indicator 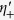, we can obtain an expression of 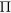 in the form of separated variables as


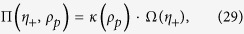


where 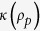 is independent of the change in 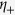, and 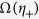 is independent of 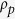. The consequence of Eq. (29) is remarkable, since it defines in the parameter space 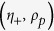 a function 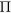 in the form of separated variables which, as compared with the original quantity 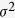, not only simplifies the description but also gives a more intuitive picture of the system behavior. Specifically, for the MG dynamics, the influences of various factors on the variance 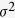 or 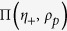 can be classified into two parts: (I) the function 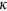 that reflects the effects of the pinning fraction 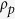 and the network structure among agents (in terms of the degree distribution 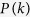, the average degree 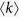, and the scaling exponent *γ*), and (II) the function Ω that characterizes the impact of the pinning pattern indicator 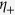 and the response of agents to resource capacities 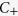 and 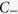 through 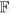. Figure 8(a,b) show the values of 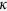 as a function of 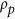 for 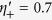 and 0.8, respectively, whereas Fig. 8(c) shows 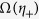 for several values of 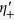. From Eqs (24) and (26), we see that Ω is a quadratic function of 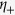 with the symmetry axis at 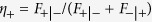, which depends on the setting of response function 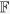. The second derivative of the function depends on 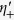.

From the definition in Eq. (25), the variance of the system for arbitrary values of 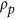 and 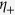 can be obtained as


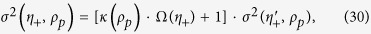


where 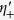 specifies the reference pinning pattern. Once we have the two respective 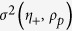 curves for the two specific pinning patterns as specified by 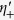 and 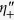, 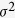 in the whole parameter space 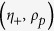 can be calculated accordingly. In particular, the quantities 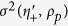 and 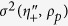 serve as a *holographic* representation of the dynamical behavior of the system in the whole parameter space. In particular, one can first obtain 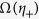 from Eqs (17) and (26), and then calculate 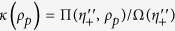, and finally obtain the value of 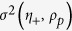 by substituting 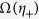 and 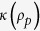 into Eq. (30).

### Analysis of Gini index

**Figure 9:**
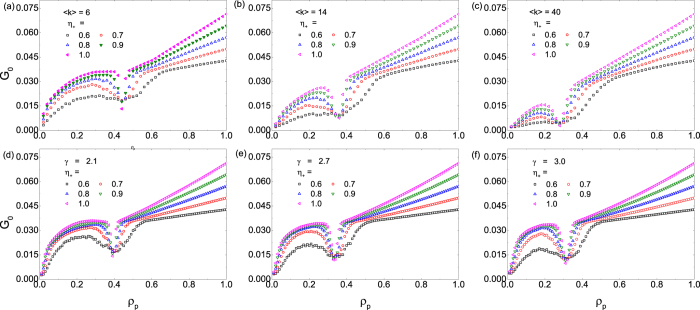
Gini index *G*_0_ as a function of the pinning fraction *ρ*_*p*_. (**a**–**c**) Results obtained from scale-free networks with degree scaling exponent 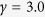
54, system size 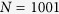, and average degree 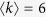, 14 and 40, respectively. (**d**–**f**) Results from scale-free networks^55^ of size 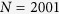 and degree scaling exponent 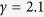, 2.7, and 3.0, respectively. The value of the pinning pattern indicator 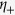 ranges from 0.6 to 1.0.

The equality of wealth is also an important criterion to assess the performance of a resource allocation system, which can be characterized by the Gini index. For MG systems without control, it was found that inequality in wealth can be pronounced when the resource utility is optimized^63^. We calculate the Gini index to uncover the interplay between pinning control and wealth equality in Boolean systems. In particular, the Gini index is defined as


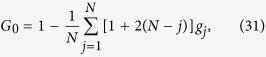


where *N* is the total number of agents in the system and 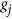 is the ratio of the wealth earned by agent *j* over the total amount of wealth in the whole system. Note that 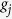 is ranked in the ascending order as 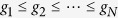. During each round of the game (each time step), the wealth of each minority agent is set to increase by one unit, while the wealth of the majority agents is unchanged. The accumulated wealth of each agent over a long time interval (e.g., 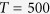 time steps) can be used to calculate the Gini index of the system according to its definition. Figure 9 shows the value of the Gini index 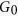 as a function of 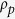, where panels (a–c) are the results for scale-free networks^54^ of scaling exponent 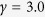 and system size 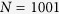, and for three different values of the average degree: 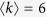, 14 and 40, respectively. Results for scale-free networks^55^ of size 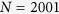 and three different values of the scaling exponent: 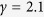, 2.7, and 3.0, are shown in panels (d–f), respectively. In each panel, the value of the pinning pattern indicator 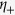 ranges from 0.6 to 1.0. In reference to the variance 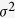 in Figs 1 and 2 for the same networks under identical dynamical parameter setting, we see that the value of 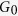 reaches a local minimum at the optimal pinning fraction 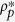. This implies that optimal use of resources and equality in wealth in a population can be realized simultaneously through pinning control.

As shown in each panel of Fig. 9, for larger values of 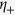 (i.e., larger biases in pinning), the value of 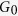 is generally larger and more sensitive to changes in the pinning fraction 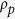, i.e., 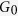 varies more rapidly with 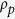. When the system’s utilization of resource is optimized at 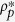, we have 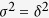 (because 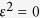 - see Eq. (5) and discussions). We see that the Gini index can be determined through the fluctuation 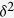 of 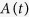. As a result, if the pinning scheme is more biased (a larger value of 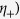, the fluctuations of 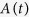 are smaller, leading to a smaller value of 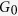. In addition, for the scale-free networks with larger average degree 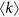, 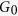 increases more rapidly as 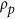 is increased from zero.

## Discussions

The phenomenon of herding is ubiquitous in social and economical systems. Herding behavior may play a positive role in certain types of dynamical processes, with examples such as promoting cooperation in evolutionary game dynamics^64^,^65^,^66^ and encouraging vaccination to prevent or suppress epidemic spreading^67^. However, in systems that involve and/or rely on fair resource allocation, the emergence of herding behavior is undesirable, as in such a state a vast majority of the individuals in the system share only a few resources, a precursor of system collapse at a global scale. A generic manifestation of herding behavior is relatively large fluctuations in the dynamical variables of the system such as the numbers of individuals sharing certain resources. It is thus desirable to develop effective control methods to suppress herding. An existing and powerful mathematical framework to model and understand the herding behavior is minority games. Investigating control of herding in the MG framework may provide useful insights into developing more realistic control method for real-world systems.

Built upon our previous works in MG systems^4^,^11^, in this paper we articulate, test, and analyze a general pinning scheme to control herding behavior in MG systems. A striking finding is the universal existence of an optimal pinning fraction that minimizes the variance and realizes the equality among the agents in the system, regardless of system details such as the degree of homogeneity of the resource capacities, topology and structures of the underlying network, and different patterns of pinning. This means that, generally, the efficiency of the system can be optimized for some relatively small pinning fraction. Employing the mean-field approach, we develop a detailed theory to understand and predict the dynamics of the MG system subject to pinning control, for various network topologies and pinning schemes. The key observation underlying our theory is the two factors contributing to the system fluctuations: intrinsic dynamical fluctuations and systematic deviation of agents’ expected attendance from resource capacity. The theoretically predicted fluctuations (quantified by the system variance) agree with those from direct simulation. In particular, in the large degree limit, for a variety of combinations of the network and pinning parameters, the numerical results approach those predicted from our mean field theory. Our theory also correctly predicts the optimal pinning fraction for various system and control settings.

In real world systems in which resource allocation is an important component, resource capacities and agent interactions can be diverse and time dependent. To develop MG model to understand the effects of diversity and time dependence on herding dynamics, and to exploit the understanding to develop pinning control methods to suppress or eliminate herding are open issues at the present. Furthermore, implementation of pinning control in real systems may be associated with incentive policies that provide compensations or rewards to the pinned agents. How to reduce the optimal pinning fraction then becomes an interesting issue. Our results provide insights and represent a step toward the goal of designing highly stable and efficient resource allocation systems.

## Additional Information

**How to cite this article**: Zhang, J.-Q. *et al.* Controlling herding in minority game systems. *Sci. Rep.* **6**, 20925; doi: 10.1038/srep20925 (2016).
